# The Spatiotemporal Dynamics of Land Use Land Cover Change, and Its Impact on Soil Erosion in Tagaw Watershed, Blue Nile Basin, Ethiopia

**DOI:** 10.1002/gch2.202000109

**Published:** 2021-05-07

**Authors:** Tadele Melese, Abebe Senamaw, Tatek Belay, Getachew Bayable

**Affiliations:** ^1^ Department of Natural Resource Management College of Agriculture and Environmental Sciences Bahir Dar University P.O. Box 5501 Bahir Dar Ethiopia; ^2^ Department of Geography and Environmental Studies College of Social Science Debre Tabor University P.O. Box 273, Debre Tabor Ethiopia

**Keywords:** land use land cover, RUSLE, soil erosion hazard, Tagaw watershed

## Abstract

The Blue Nile basin is one of the hot‐spots of soil erosion areas in Ethiopia. However, the impact of land use changes on soil erosion is poorly understood in the Tagaw watershed. Hence, the objective of the study is to assess the impact of land use changes on soil erosion in Tagaw watershed over the last 31 years. Rainfall, soil, satellite images and topographic data are acquired from field survey and secondary sources. A Revised Universal Soil Loss Equation (RUSLE) model is used to estimate soil erosion. The mean annual and total potential soil losses of the watershed are 19.3, 22.9, 26 and 0.06–503.56, 0.11–516.67, and 0.00–543.5 tons ha^−1^ yr^−1^ for 1995, 2006 and 2016 respectively. The highest soil loss is found for bare land. The RUSLE model further showed that the highest soil erosion occurred in 2016 whereas the lowest soil erosion occurred in 1995.

## Graphical abstract

The impact of land use land cover changes on soil erosion over the last 31 years are assessed by integrating geospatial technology and a revised universal soil loss equation model. The results reveals a high rate of land use land cover changes, which have a significant impact on soil erosion in the study area.

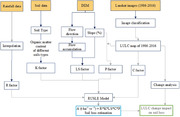

## 1 Introduction

Changes in land use land cover (LULC) have become a global concern.^[^ ^1^ ^]^ According to the report,^[^ ^2^ ^]^ LULC changes are the major environmental challenges in various parts of the world. Currently, land use land cover changes have been recognized universally as a fundamental driver of global environmental change.^[^ ^3^ ^]^ Globally, there had been an increase in crop and pasture land at the expense of forest land, savanna land, and natural grassland.^[^ ^4^, ^5^ ^]^ Land use land cover changes contribute significantly to earth atmosphere interaction, climate change, loss of biodiversity,^[^ ^6^, ^7^, ^8^ ^]^ forest fragmentation, the modification and conversion of bird community,^[^ ^9^ ^]^ soil resource,^[^ ^7^, ^10^, ^11^, ^12^, ^13^, ^14^, ^15^ ^]^ groundwater recharge, and coffee production.^[^ ^6^, ^7^, ^8^, ^9^, ^10^ ^]^

In Ethiopia, land use land cover change is a common phenomenon. There is a high rate of land conversion from forest land, bush shrub, and grassland to cultivated and built‐up areas.^[^ ^11^, ^12^, ^13^ ^]^ Change has contributed to soil degradation in the form of soil erosion.^[^ ^10^, ^14^ ^–^ ^17^ ^]^ Soil erosion is a serious environmental problem and is severely influenced by land use land cover change.^[^ ^18^, ^19^, ^20^ ^]^ There was a high rate of soil erosion in cultivated land than other land cover types.^[^ ^18^, ^21^, ^22^ ^]^ This results in a decline in agricultural productivity, which forces farmers to look for new fertile land causing expansion of cultivated land at the expense of the forest ecosystem.^[^ ^33^, ^34^ ^]^ In the highland of Ethiopia, a significant portion of cultivated land has been out of production every year due to soil erosion and land degradation.^[^ ^35^ ^]^

Tagaw watershed is well known in agricultural production in the Blue Nile basin targeted by the government. However, it is very vulnerable to soil erosion due to its undulating topography, extreme deforestation, burning of crop residue, rugged topography, inappropriate land management practice, and historic settlement.^[^ ^32^ ^]^ Assessment of soil erosion hazard and its impact in a spatially distributed is essential at the watershed scale to implement sound conservation efforts and natural resource management^[^ ^26^, ^27^, ^28^ ^]^ and land use planning project.^[^ ^25^, ^37^ ^]^ However, there was very scanty information on the impact of LULC change on soil erosion in the study area. Therefore, the central objective of this work was to assess the impact of land use land cover change on soil erosion in Tagaw watershed in the Blue Nile basin. The specific objectives of the study were: 1) examine LULC change between 1986 and 2016, 2) estimate the actual soil loss for the Tagaw watershed using revised universal soil loss equation (RUSLE), and 3) assess the differences in the proportion of the area of LULC classes in the erosion risk maps.

A wide variety of models are available for assessing soil erosion risk. Many soil erosion models have been developed concerning the estimation and the prediction of soil loss and degradation. Estimation of soil erosion consists of physical‐based models like Water Erosion Prediction Project,^[^ ^29^, ^30^ ^]^ European Soil Erosion Soil Model,^[^ ^31^, ^32^, ^33^ ^]^ and empirical‐based models like universal soil loss equation (USLE).^[^ ^44^, ^47^ ^]^ The USLE and its revised version RUSLE are two of the empirical models that have been most commonly used, and RUSLE is an empirically based model that can estimate and forecast the long‐term average annual rate of soil erosion.^[^ ^31^, ^35^, ^36^ ^]^ The RUSLE model in the geographic information system (GIS) environment can predict erosion potential on a cell‐by‐cell basis,^[^ ^23^, ^24^, ^25^ ^]^ which is effective when attempting to identify the spatial pattern of soil loss present within a large watershed area used in this study.

## 2 Experimental Section

### 2.1 Description of Study Area

**Figure 1:**
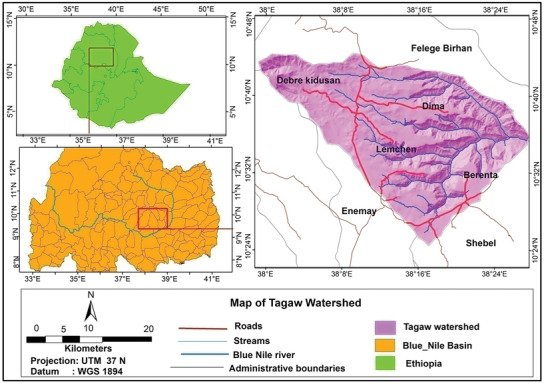
Location map of Tagaw watershed.

The astronomical location of Tagaw watershed extends from 10° 22′ 20″ N to 10° 42″ N latitude and 38° 2′ E to 38° 27′ E longitude in the northern highlands of Ethiopia (**Figure** **1**). It covers an area of 960 km^2^. The annual rainfall of the study area is around 1538.4 mm. The mean annual maximum and minimum temperatures in the study area is 23.3 and 7 °C, respectively. The major soil types in the study watershed are Eutric Vertisols (59.3%) and Eutric Leptosols (37.6%). The altitudinal variation of the study area generally ranges from 1149 to 3742 m above the sea level. The mixed farming system is the dominant economic activity and the main source of the livelihood of the population.

### 2.2 Data Sources and Image Processing

This study had used two kinds of data: primary data and secondary data. Ground control points were collected using Garmin Global Position System (GPS). The field data collection was done to verify the classified image and to collect the necessary land use land cover data for training area delineation and accuracy assessment. The analysis of land use land cover change was carried out using Landsat images. The Landsat images were acquired freely from United States Geological Survey (USGS) website (www.earthexplorer.usgs.gov). In this study, Landsat‐5 TM, Landsat‐7 ETM^+^, and Landsat‐8 OLR and TIR imageries were used for image analysis. All of the imageries were considered to be in the dry season during the February month. ASTER Global Digital Elevation Model (DEM) data with 30 m spatial resolution were also used to generate slope, aspect, and watershed delineation using Arc GIS environment.

### 2.3 Methods of Analysis

Image classifications were conducted by using the maximum likelihood algoritchm of the supervised classification method. Land use and land cover types or classified images were used as inputs for generating crop management (*C*) factor and support practice (*P*) factor of the revised universal soil loss equation. The RUSLE framed with GIS and remote‐sensing techniques were used to estimate the mean annual soil loss that occurred in Tagaw watershed. The complete methodology involved the use of the RUSLE in a GIS environment, with factors obtained from meteorological stations, soil surveys, satellite images, digital elevation model, and results of other relevant studies. Individual GIS layers were built for each factor in the RUSLE and combined by cell‐grid modeling procedures in Arc GIS to predict soil loss in a spatial field. Finally, obtained soil loss result was classified as presented in **Table** **1**.

**Table 1:** Erosion classes and rates with indicators

Code	Classes	Erosion rate [tons ha^−1^ yr^−1^]	Indicators
1	Very slight	0–2	No evidence of compaction or crusting of the soil; no wash marks or scour features; no splash pedestals or exposure of tree routes; over 70% plant cover (ground and canopy)
2	Slight	2–5	Some crusting of soil surface; localized wash but no or minor scouring; rills every 50 to 100 m; small splash pedestals; 1–5 mm depth; where stones or exposed trees protect underlying soil; occupying not more than 10% of the area; soil level slightly on unslope or windward sides of plants and boulders; 30–70% plant cover
3	Moderate	5—10	Wash marks; discontinuous rills spaced every 20–50 m; splash pedestals and exposed tree roots mark the level of the former surface, soil mounds protected by vegetation, all the depths of 5–10 mm and occupying not more than 10% of the area, slight to moderate surface crusting; 30–70% plant cover; slight risk of pollution problems down streams if slopes discharge straight into watercourses
4	High	10–50	Connected and continuous network rills every 5–10 m or gullies spaced every 5–10 m tree route exposure, splash pedestals, and soil mounds to depths of 10–50 mm occupying not more than 10% of the area; crusting of the surface cover large areas; <30% land cover danger of pollution and sedimentation problems down streams
5	Severe	50–100	Continuous network rills every 2–5 m or gullies every 20 m; tree route exposure; splash pedestals and soil mounds to depths of 50–100 mm occupying not more than 10% of the area; splays of coarse materials; bare soil; siltation of bodies damage to roads by erosion and sedimentation
6	Very severe	100–500	A continuous network of channels with gullies every 5–10 m, surrounding soil heavy crusted; severe siltation; pollution and eutrophication problems: bare soil
7	Catastrophic	>500	Extensive networks of rills and gullies; large gullies (>100 m^2^) every 20 m; most of the original soil surface removed; severe damage from erosion and sedimentation on‐site and downstream

## 3 Results

### 3.1 Image Classification

The classified maps of the study area were produced for each of the years in 1986, 1996, 2006, and 2016. The accuracy assessment was performed using the confusion matrix. The overall accuracy values for the maps of 1986, 1995, 2006, and 2016 were 87%, 85%, 86.5%, and 89%, respectively (see **Table** **2**).

**Table 2:** The overall accuracy assessment of classifications

Class names	Accuracy results [%]							
	1986		1995		2006		2016	
	Producers	Users	Producers	Users	Producers	Users	Producers	Users
Farmland	79	75	69	79	65	69	89	90
Built‐up	82	87	96	84	98	64	82	67
Grassland	55	50	57	74	59	98	100	87
Marshland	80	67	64	79	100	64	100	62
Bare land	92	99	74	98	72	78	82	78
Forest	75	80	95	79	89	76	100	93
Shrubland	61	89	66	86	78	57	98	84
Overall classification	87%		85%		86.5%		89%	

### 3.2 LULC Analysis

The LULC for the study area between the two successive years were assessed carefully. The overall change in LULC over the last three decades was also assessed. As indicated in **Table** **3**, the area coverage of farmland, built‐up, and bare land was increased from 1986 to 2016, whereas the area coverage of grassland, forests, marshland, and shrubland was decreased. However, the area coverage of forests was increased from 2006 to 2016.

**Table 3:** LULCC conversion matrix between 1986 and 2016 (FL: Farmland, BU: Built‐up, BL: Bare land, F: Forest, GL: Grassland, ML: Marshland, SL: Shrubland)

Land cover1986		Land cover 2016							
		BL	BU	FL	F	GL	ML	SL	*GT*
	BL	12 823	986	1675	0	279	342	287	16 392
	BU	197	14 861	1747	23	948	1249	1258	20 283
	FL	92	1010	15 180	134	1848	1357	1290	20 911
	F	1490	483	433	1873	142	117	267	4805
	GL	743	12	678	1492	4215	1642	820	9602
	ML	395	651	58	961	901	726	5011	8703
	SL	1357	3101	2017	1923	968	2969	2969	15 304
	GT	17 097	21 104	21 788	6406	9301	8402	11 902	96 000

This was just the general impression of land cover dynamics based on the comparison of individual land cover types. The farmland and built‐ups portrayed an increasing pattern of change during the period comparisons between 1986 and 2016. It increased from 16 012 to 21 788 ha and from 13 986 to 21 104 ha in 1986 and 2016, respectively. The area coverage of bare land, built‐up, and farmlands was increased significantly from 1986 to 2016, whereas shrubland area coverage was significantly decreased (**Table** **4**).

**Table 4:** LULC types and areas covered by the respective land‐use types

Class names	Area covered by respective land‐use/cover type							
	1986		1995		2006		2016	
	Area [ha]	[%]	Area [ha]	[%]	Area [ha]	[%]	Area [ha]	[%]
FL	16 012	16.7	18 703	19.5	20 911	21.8	21 788	22.7
BU	13 986	14.6	16 197	16.9	20 283	21.1	21 104	22
GL	11 002	11.5	9986	10.4	9602	10	9301	9.7
ML	7810	8.1	9007	9.4	8703	9.1	8402	8.8
BL	12 990	13.5	14 503	15.1	16 392	17.1	17 097	17.8
F	9177	9.6	6904	7.2	4805	5	6406	6.7
SL	25 023	26	20 700	21.6	15 304	15.9	11 902	12.4
Total	96 000	100	96 000	100	96 000	100	96 000	100

It can be summarized from Table 4 that in 2016 shrubland and the forest area significantly decreased to 52.4% and 30.2%, respectively, when compared to the shrubland and the forest area in 1986, while the grassland area slightly decreased by 15.5% and marshland area decreased by 7.6%. As the same table indicated in 2016, the built‐up, farmland, and bare land were significantly increased to 50.9%, 36.1%, and 31.6%, respectively, when compared to the built‐up, farmland, and bare land in 1986. Generally, during comparisons among 1986, 1995, 2006, and 2016, the farmland and built‐ups were increased with the rates of 1.16% and 1.64% per year. Bare land also increased with a rate of 1.02%. In contrast, the shrubland and forest decreased with the rates of 1.96% and 0.97% per year, respectively, between the years 1986 and 2016.

The area of land that was occupied by bare land, farmland, and built‐up in 1986 and 2016 took the vast portion in terms of area coverage in the study area. The bare land, farmland, and built‐up (Table 3) were converted mainly from grasslands, shrubland, forests, and marshland. The study showed that bare land coverage increased significantly between 1986 and 2016. The same is true for the land cover of built‐up and farmlands. Meanwhile, shrubland was significantly decreased in the area between 1986 and 2016 (**Table** **5**).

**Table 5:** Rates and LULC changes in the Tagaw watershed

Land use class	Change in land use area [ha and %] coverage; gain (+) or loss (−)								Rate of change [%]
	1986–1995		1995–2006		2006–2016		1986–2016		
	Area [ha]	[%]	Area [ha]	[%]	Area [ha]	[%]	Area [ha]	[%]	1986–2016
FL	2691	16.8	2208	11.8	877	4.2	5776	36.1	1.16
BU	2211	15.8	4086	25.2	821	4.0	7118	50.9	1.64
GL	−1016	−9.2	−384	−3.8	−301	−3.1	−1701	−15.5	−0.50
ML	1197	15.3	−304	−3.4	−301	−3.5	592	−7.6	−0.24
BL	1513	11.6	1889	13.0	705	4.3	4107	31.6	1.02
F	−2273	−24.8	−2099	−30.4	1601	33.3	−2771	−30.2	−0.97
SL	−4323	−17.3	−5396	−26.1	−3402	−22.2	−13 121	−52.4	−1.69

### 3.3 Estimated Soil Loss and Mapping

**Figure 2:**
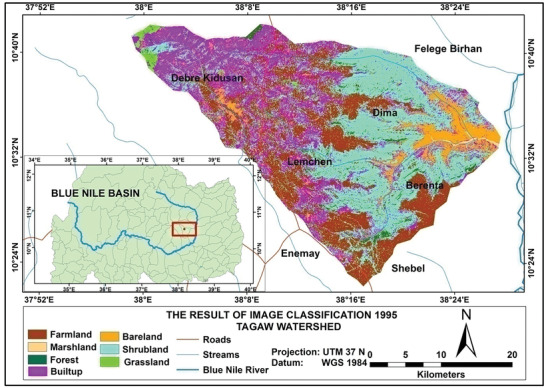
Land use/land cover map of the study area for 1995.

**Figure 3:**
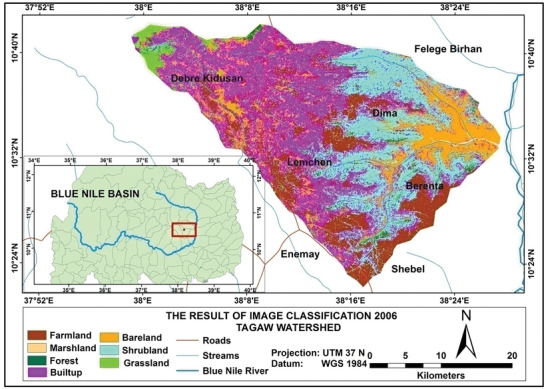
Land use/land cover map of the study area for 2006.

**Figure 4:**
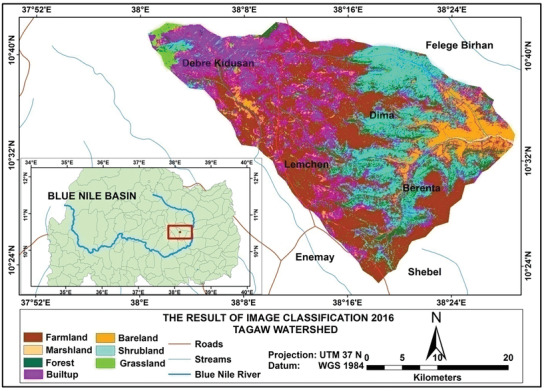
Land use/land cover map of the study area for 2016.

Based on the analysis, the total amounts of soil loss in Tagaw watershed are about 950 039, 1012 196, and 1123 284 tons yr^−1^ in the years 1995, 2006, and 2016 (see **Figures** **2**–**4**), respectively. Besides the mean annual soil losses of Tagaw watershed are 19.32, 23.567, and 25.341 tons ha^−1^ yr^−1^ in 1995, 2006, and 2016, respectively. As indicated in **Table** **6**, the amounts of soil loss of each parcel of land in the watershed range from 0.06 to 503.56, from 0.00 to 516.67, and from 0.11 to 543.5 tons ha^−1^ yr^−1^ in 1995, 2006, and 2016, respectively.

**Table 6:** Soil loss in different years

Result description	Soil loss in different years [tons ha^−1^ yr^−1^]		
	1995	2006	2016
Soil loss range	0.06–503.56	0.00–516.67	0.11–543.5
Total annual soil loss	950 039	1012 196	1123 284
Mean soil loss	19.3	22.93	24.3214

**Figure 5:**
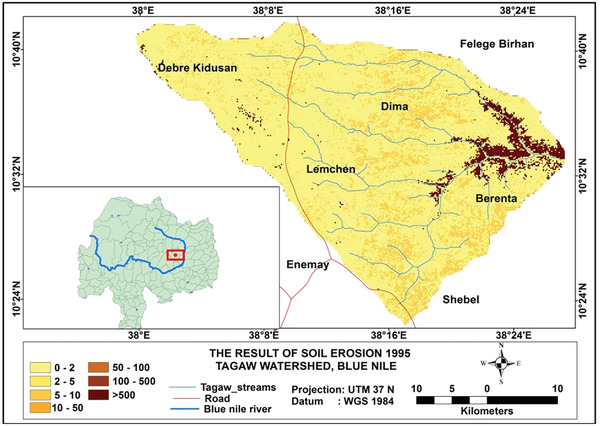
Map of soil erosion in 1995.

This study employed^[^ ^73^ ^]^ soil erosion class mapping scale to effectively visualize the spatial distribution of soil erosion hotspot areas for soil erosion. According to ref. ^[^ ^73^ ^]^, the soil erosion level was classified into seven classes (tons ha^−1^ yr^−1^): very slight (0–2), slight (2–5), moderate (5–10), high (10–50), severe (50–100), very severe (100–500), and catastrophic (>500), respectively. As presented in **Figures** **5** **–** **7** and **Table** **7**, the extent of soil erosion ranges from insignificant erosion to 503.56 tons ha^−1^ yr^−1^, especially in the year 1995, and very slight, slight, and moderate soil erosion risk categories occupied with ≈65.22% of the total area. However, the areas of moderate, high, severe, and especially catastrophic soil erosion categories strongly increased in 2006 and 2016.

**Figure 6:**
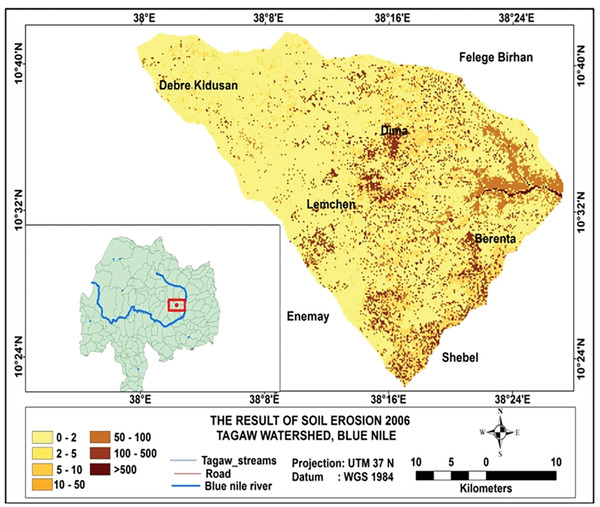
Map of soil erosion in 2006.

**Figure 7:**
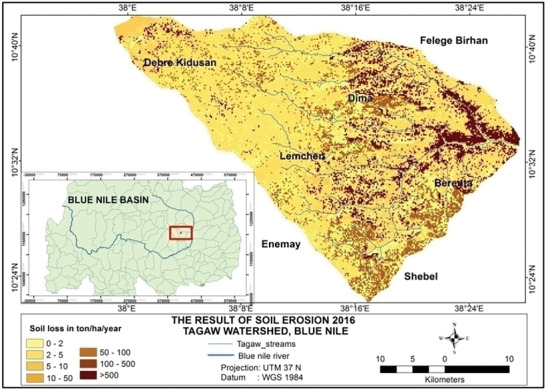
Map of soil erosion in 2016.

**Table 7:** Soil erosion classification based on Morgan^[^ ^73^ ^]^

Erosion rate [tons ha^−1^ yr^−1^]	Erosion classes	Coverage of the erosion affected area [%]		
		1995	2006	2016
0–2	Very slight	26.04	27.6	19.4
2–5	Slight	11.56	12.3	8.48
5–10	Moderate	27.62	7.17	12.3
10–50	High	13.15	24.43	28.98
50–100	Severe	12.42	16.6	13.91
100–500	Very severe	3.84	9.8	13
>500	Catastrophic	0.37	3.2	5.61

### 3.4 Effect of Land Use Land Cover Change on Soil Erosion Hazard

As described in land‐use change analysis, various LULC were found in the study area. The vulnerability was detected from the largest proportion of the high rate of the land‐use type. A high rate of soil erosion means that the material in such a land‐use type is more detachable for several reasons.

The assessment of soil loss for all land‐use types was calculated in percentage for each soil loss range. From the analysis, the susceptible land uses on soil erosion were bare land, agriculture, and settlement. This is because most of the soil erosion occurrences were dominated by these land‐use types. For instance, in 1995, 28%, 21%, and 22% of the erosion ranging from 100 to 500 tons ha^−1^ yr^−1^ occurred in bare land, farmland, and settlement relative to other land‐use types, respectively (**Table** **8** **)**. Meanwhile, forest and grassland were the two least erosion sources in the study catchment in the same range at the same time.

**Table 8:** Erosion coverage area of 1995, 2006, and 2016 by land‐use types

Erosion [tons ha^−1^ yr^−1^]	Erosion coverage area 1995 by land‐use types [%]						
	Forest	Built‐up	Grassland	Marshland	Bare land	Farmland	Shrubland
0–2	0	21	9	13	26	24	7
2–5	2	19	11	9	23	24	12
5–10	2	18	12	8	24	24	12
10–50	2	26	9	7	25	23	8
50–100	3	23	8	13	24	23	6
100–500	3	22	5	13	28	21	7
>500	2	25	4	6	31	26	6
Erosion [tons ha^−1^ yr^−1^]	Erosion coverage area 2006 by land‐use types [%]						
	Forest	Built‐up	Grassland	Marshland	Bare land	Farmland	Shrubland
0–2	2	21	9	13	26	24	7
2–5	2	19	11	9	23	24	12
5–10	3	24	9	13	24	22	7.5
10–50	2	26	9	7	25	23	8
50–100	3	23	8	13	24	23	6
100–500	2	24	4	13	29	23	5
>500	1	27	3	4	32	28	5
Erosion [tons ha^−1^ yr^−1^]	Erosion coverage area 2016 by land‐use types [%]						
	Forest	Built‐up	Grassland	Marshland	Bare land	Farmland	Shrubland
0–2	2	20	10	12	24	22	11
2–5	3	24	11	9	23	19	12
5–10	2	24	9	12	24	22	7
10–50	4	23	8	7	25	24	9
50–100	3	23	7	14	24	23	6
100–500	1	27	2	8	32	28	4
>500	1	29	2	3	33	28	4

Soil loss for farmland and bare land in the study periods was portrayed as a great change. As shown in **Table** **9**, during the two study periods, the trend of soil loss in the three study years also indicates that a sharp increase for shrubland, marshland, grassland, and built‐up areas, while there is a declining trend in forest covers between the year 2006 and 2016. This is because the coverage of forest was increased at a slow rate. The major source of increased erosion in the study watershed is the bare land followed by farmland. The mean annual soil erosion in bare land was 29 tons ha^−1^ yr^−1^ in 1995, and this value was increased by 39.6 tons ha^−1^ yr^−1^ in 2016 (Table 9). Increased soil erosion in bare land was not surprising. This is because the vast portion of the area was not covered by vegetation covers and exposed to soil erosion. In the case of farmland area, the mean annual soil losses were 24.6 and 34.25 tons ha^−1^ yr^−1^ in 1995 and 2016, respectively. The mean annual soil losses of shrubland were 14.6, 19.2, and 22.31 tons ha^−1^ yr^−1^ in 1995, 2006, and 2016, respectively. The change of soil loss for shrubland is about 7.71 tons ha^−1^ yr^−1^. The study suggested that increasing the area of bare land has a great influence on soil erosion than other land covers, even strong than farmland and reduction of forest cover result. As indicated in the table, farmland and shrubland have a great impact on soil erosion.

**Table 9:** Mean annual soil loss from each land use land cover types

Class names	Mean annual soil loss [tons ha^−1^ yr^−1^]			Change of soil loss [tons ha^−1^ yr^−1^]		
	1995	2006	2016	1995–2006	2006–2016	1995–2016
Farmland	24.6	31.23	34.25	6.63	3.02	9.65
Built‐up	22	26.5	28	4.5	1.5	6
Grassland	17.2	19	19.5	1.8	0.5	2.3
Marshland	18.3	23	23.5	4.7	0.5	5.2
Bare land	29	33.98	39.6	4.98	5.62	10.6
Forest	9.4	7.6	8.34	−1.8	0.74	−1.06
Shrubland	14.6	19.2	22.31	4.6	3.11	7.71

**Table** **10** revealed that the farmland, bare land, and built‐up could be strongly affected by erosion risk. For instance, bare land was the fourth largest in the study area, but the amount of soil loss was highest among all land‐use categories in 1995. The total soil losses of farmland, bare land, and built‐up were 296 139, 432 867.34, and 235 745.82 tons yr^−1^ in 2016, respectively.

**Table 10:** The estimate of soil loss at different land uses with the respective area

Class names	Years					
	1995		2006		2016	
	Area [ha]	Soil loss [tons yr^−1^]	Area [ha]	Soil loss [tons yr^−1^]	Area [ha]	Soil loss [tons yr^−1^]
Farmland	18 700	243 463	20 900	259 456	21 800	296 139
Built‐up	16 200	197 506.91	20 300	201 342.32	21 100	235 745.82
Grassland	10 000	34 568	9600	44 087	9300	45 376
Marshland	9000	45 789	8700	46 471	8400	51 800
Bare land	14 500	381 345.98	16 400	406 349.23	17 100	432 867.34
Forest	6900	7892.33	4800	11 563.93	6400	7572.435
Shrubland	20 700	39 473.60	15 300	42 926.71	11 900	53 783.78

## 4 Discussion

### 4.1 Land Use Land Cover Change Analysis

To demonstrate spatial and temporal trends of land use land cover change and to make useful discussion, land use land cover change analysis is done.^[^ ^25^, ^38^, ^48^ ^]^ In this regard, in the highland of Ethiopia there is an expansion of cultivated and built‐up areas at the expense of vegetation cover.^[^ ^39^, ^40^, ^41^, ^42^ ^]^ The result of this study also revealed that, in the last three decades, the high rate of land use land cover change, particularly, farmland, built‐up area, shrubland, forest land, and grazing, was observed. This result is concurrent with the results of a previous study in Ethiopia.^[^ ^43^, ^44^, ^45^, ^46^ ^]^ According to ref. ^[^ ^57^ ^]^, finding in Gelda catchment, Lake Tana watershed Ethiopia for the period 1957–2014, and, as in ref. ^[^ ^58^ ^]^, Lake Wenchi watershed central highland of Ethiopia for the period of 1973–2017 also reported a similar trend of land use land cover change.

According to the result over the last 3 years, there was a degradation of shrub and forest lands, as shown in Table 3; 1490,483, and 433 ha of forest land were converted into bare land, built‐up area, and farmland, respectively. Similarly,1357, 3101, and 2017 ha of shrubland were converted into bare land, built‐up area, and farmland, respectively. This result is consistent with those of authors,^[^ ^39^, ^44^, ^46^, ^49^, ^50^ ^]^ who reported that vegetation areas were change to cultivated and built‐up areas at different periods. This implies that there is destruction in natural forests in search of farm plots, construction material, and built‐up areas in the study watershed. In contrast, refs. ^[^ ^51^, ^61^ ^]^ states that cropland decreased by 9% while grassland and vegetation cover increase by 136% and 96%, respectively, from the period of 2010 to 2015 in Melka watershed highland Ethiopia. As shown in Table 5, expansion of bare land shows the impact of unsustainable utilization of grass, forest, and shrublands. Expansion of bare and cultivated lands at the expense of natural vegetation has intensified the problem of land degradation through soil erosion by water.^[^ ^52^, ^53^, ^54^ ^]^

### 4.2 Effect of Land Use Land Cover Change on Soil Erosion

Land use land cover change is one of the triggering factors for the occurrence of soil erosion hazards.^[^ ^65^ ^]^ According to ref. ^[^ ^66^ ^]^, physical factors are essential for land‐use planners in spatial planning. Hence, the integration of spatial and temporal variation of land use land cover and soil erosion hazard map is critical for land‐use planning.^[^ ^55^, ^57^, ^58^ ^]^

In Ethiopia, the land use land cover change played a significant role in an increase in the rate of soil erosion hazard.^[^ ^55^, ^56^, ^77^ ^]^ According to the estimation of refs. ^[^ ^34^, ^71^ ^]^, the annual soil loss of highlands of Ethiopia ranges from 1248 to 23 400 million tons per year from 78 million hectares (16–300 tons ha^−1^ yr^−1^) of pasture, ranges, and cultivated fields throughout Ethiopia. The average annual rate of soil loss in Ethiopia is estimated to be 12 tons ha^−1^ yr^−1^, and it can be even higher on steep slopes with soil loss rates being greater than 300 tons ha^−1^ yr^−1^ or 250 mm yr^−1^, where vegetation covers are scant.^[^ ^72^ ^]^ The result of this study also shows that mean annual soil loss gradually increases in the study period, which shows 19.32, 23.0, and 24.3 tons ha^−1^ yr^−1^ in the years 1995, 2006, and 2016, respectively.

Bare land and farmland were the most susceptible to soil erosion than other types of land uses.^[^ ^60^, ^61^, ^62^, ^63^ ^]^ This is because the two land‐use types have less vegetation cover than others.^[^ ^18^, ^22^, ^64^ ^]^ As shown in Table 7, 28%, 29%, and 32% of bare lands were affected by very severe soil erosion in the years 1995, 2006, and 2016, respectively. Similarly, 21%, 23%, and 28% of cultivated land were vulnerable to very severe soil erosion classes in the years 1995, 2006, and 2016, respectively. Likewise, ref. ^[^ ^78^ ^]^ also reported that expansion of cultivation practices has increased the mean annual soil loss rate by 16.3 tons ha^−1^ yr^−1^. However, less than 5% of forest and grassland were affected by a very severe erosion class. Vegetation cover is very prominent in determining soil erosion by water and effective in controlling runoff effects on soil erosion.^[^ ^31^, ^59^, ^66^ ^]^ The grassland showed a small‐area coverage of erosion intensity because of its protective potential in runoff and raindrop. The study suggested that increasing the area of bare land has a great influence on soil erosion than other land‐cover types.

The spatial distribution of soil loss in the watershed varied across LULC types. The eastern part of the study area is affected by a very severe soil erosion class, which is dominated by bare land and farmland (Figures 5, 6, 7).

The positions of grassland that lie in gentle slopes do not aggravate the occurrence of high soil erosion. Generally, the soil loss rate is the highest in bare land, farmland, and built‐up in all erosion classes. During the year 2006, the vulnerable/susceptible land uses on soil erosion were similar to the analysis of 1995. Those land uses were bare land, settlement, and agriculture. The only difference was that the percentage of soil loss areal coverage increased from the year 1995 to 2006. For instance, in 2006, 29%, 23%, and 24% of the erosion ranging from 100 to 500 tons ha^−1^ yr^−1^ occurred in bare land and agriculture, respectively (Table 8).

As explained, Table 8 shows the proportion of soil erosion in each land‐use type in the study catchment. Bare land, farmland, and settlements were highly exposed to soil erosion. Most of the soil erosion occurrences were dominated by bare land and agriculture from other land‐use types. For instance, during this year, 32% and 28% of the erosion ranging from 100 to 500 tons ha^−1^ yr^−1^, and 24% and 22% of the erosion ranging from 0 to 2 tons ha^−1^ yr^−1^ occurred in bare land and agriculture, respectively. Forest and grassland were the two least erosion sources in the study watershed with soil erosion values of 2% and 6% ranging from 100 to 500 tons ha^−1^ yr^−1^ like the previous results.

As shown in Table 10, farmland, bare land, and built‐up areas were strongly affected by soil erosion hazards. Soil losses of farmland, bare land, and built‐up were 296 139, 432 867.34, and 235 745.82 tons yr^−1^ in 2016, respectively. The farmland is the second highest soil loss next to bare land in 1995, 2006, and 2016 because a large area of natural forest and shrubland converted to agricultural and bare land. The studies done by refs. ^[^ ^67^, ^68^ ^]^ showed similar results. Meanwhile, shrubland, grassland, and marshland were covered by grasses and trees with small canopies; therefore, these land‐use types were considered as low soil erosion risks.^[^ ^63^, ^69^, ^79^, ^80^, ^81^ ^]^ Based on the analysis, the area of land cover has a great influence on soil erosion. As obtained from soil loss assessment, the amount of soil erosion was in the order of bare land > farmland > built‐up >shrubland > grassland > marshland > forest in 1995 and 2016. In 2006, however, the amount of soil loss was bare land > farmland > built‐up > marshland > grassland > shrubland > forest. In 2016, the area coverage was bare land > farmland > built‐up > shrubland > marshland > grassland > forest.

As explained by different studies,^[^ ^71^, ^72^, ^74^ ^]^ land‐use change percentage of the watershed strongly influences the degree of soil erosion. In this study, the three successive years of the forest, bare land, farmland, built‐up, shrubland, marshland, and grassland area coverage were taken as influencing factors to inspect the effects on soil erosion in Tagaw watershed.

As obtained from the analysis, land cover percentage of bare land, farmland, and built‐up showed a strong positive relationship with soil erosion, implying that areas of land cover are strong explanatory variables of soil erosion whereas forest cover, marshland, shrubland, and grassland were negatively related to soil erosion. Bare land was positively related to the amount of soil erosion implying that when the area of bare land of the watershed is increased across the watershed, its buffering effect becomes high and, hence, soil erosion becomes more.^[^ ^74^, ^75^ ^]^

Forest cover is one of the most important variables that strongly influence soil erosion of the watershed.^[^ ^74^, ^75^, ^76^ ^]^ The area of forest cover band soil erosion showed a negative relationship in the study watershed. This justification is in the line with the justification by ref. ^[^ ^73^ ^]^; the forest cover/vegetation is effective in controlling runoff in soil erosion. Consequently, the erosion rate in the forested area is lower than that in the less vegetated area.^[^ ^30^, ^70^ ^]^

Farmland was positively related to the amount of soil erosion implying that when the area of farmland of the watershed is increased across the watershed, its effect becomes high and, hence, soil erosion becomes more. This agreed with the previously conducted findings:^[^ ^37^, ^71^ ^]^ the high soil erosion mostly occurs in farmland areas that were more vulnerable to the collapse of an embankment and it increased soil erosion

Marshland and grassland were negatively related to soil erosion in the study period. This is consistent with the findings of refs. ^[^ ^85^, ^86^ ^]^, where the grassland also has great potential to reduce peak discharge rate and runoff, and negatively correlated. The lower runoff is the lower soil erosion that existed.^[^ ^83^, ^84^ ^]^ Built‐up was positively related to soil erosion.

## 5 Conclusions

Land use and land cover of the Tagaw watershed for the period of 1986–2016 showed significant changes over the last three decades. Based on the analysis of the input data, in general, about seven land use land cover classes were identified: bare land, farmland, forest, shrubland, built‐up, grassland, and marshland. Land cover postclassification change analysis for the periods (1986–1995, 1995–2006, 2006–2016, and 1986–2016) revealed that some important land cover changes were consistent in all subperiods. Farmland, bare land, and built‐up were increased significantly in the study periods. Meanwhile, forest, marshland, grassland, and shrubland were decreased in the same periods. However, forest coverage shows a slow increase from the period 2006 to 2016 understudy.

According to the soil erosion map of Tagaw watershed (1995, 2006, and 2016), the highest soil erosion rate occurred in 2016 whereas the lowest occurred in 1995. Moreover, different land‐use types in terms of area size and pattern influenced the soil erosion map in Tagaw watershed in1995, 2006, and 2016. Areas of bare land, farmland, and built‐up highly increased in the study years and have significant as well as strong positive relation with soil erosion. In contrast, the shrubland decreased significantly and its area size has a strong negative relationship with soil erosion.

The findings of land use land cover change over the last three decades showed that population growth, improper cultivation, uncontrolled grazing, and deforestation were the major drivers of land‐use change and have resulted in accelerating soil erosion in the study watershed. The area with smaller land coverage (forest, shrubland, marshland, and grassland) showed a higher risk of soil erosion than the larger land cover did. The area with larger land coverage (farmland, built‐up, and bare land) showed a higher risk of soil erosion than the smaller land area cover did. Generally, there is a significant impact on land use land cover change on soil erosion in Tagaw watershed. To this end, the study provides baseline information for conservation measurement and utilization in this area and offers a technical basis for using the RUSLE to estimate soil erosion and assessing the impact with corresponding LULC.

## Conflict of Interest

The authors declare no conflict of interest.

## Data Availability

Data are available in USGS website as shown in the citation.
